# Supplementary material for: Assessing the performance of multimodal large language models in experimental information extraction from liquid–liquid phase separation literature
Source: Sci Rep. 2026 Apr 10;16:16950. doi: 10.1038/s41598-026-47277-0 (PMC13230596; doi:10.1038/s41598-026-47277-0)
Supplement: Supplementary file 2 — Supplementary Material 2 [file 41598_2026_47277_MOESM2_ESM.docx]

Supporting Information

**Assessing the Performance of Multimodal Large Language Models in Experimental Information Extraction from Liquid-liquid Phase Separation Literature**

Ka Yin Chin^1^, Satoru Fujii^1^, Shoichi Ishida^1^, and Kei Terayama^1,2,3,4*^

^1^ Graduate School of Medical Life Science, Yokohama City University, 1-7-29, Suehiro-cho, Tsurumi-ku, Yokohama, Kanagawa, 230-0045, Japan.

^2^ RIKEN Center for Advanced Intelligence Project, 1-4-1, Nihonbashi, Chuo-ku, Tokyo 103-0027, Japan.

^3^ MDX Research Center for Element Strategy, Tokyo Institute of Technology, 4259 Nagatsuta-cho, Midori-ku, Yokohama, Kanagawa, 226-8501, Japan.

^4^Department of Life Science and Technology, School of Life Science and Technology, Institute of Science Tokyo, 4259 Nagatsuta-cho, Midori-ku, Yokohama, Kanagawa, 226-8501, Japan.

^*^E-mail: terayama@yokohama-cu.ac.jp

**Contents:**

Table S1. Precision scores of all proposed methods.

Table S2. Recall scores of all proposed methods.

Table S3. Results of paired Wilcoxon signed-rank tests comparing the best-performing method (Fig. + Text (C&M) with Minimal + DK + Guide) against other extraction methods of the figure-by-figure extraction.

Table S4. Average F1-scores from temperature parameter optimization for Gemini 2.5 Pro.

Table S5. Average and total extraction costs per figure for each MLLMs.

Table S6. Comparison of extraction performance between scatter plots and microscopic images.

Table S7. Correspondence between papers and reference numbers in the evaluation dataset.

Figure S1. Confusion matrices showing the counts of True Positives (TP), False Positives (FP), False Negatives (FN), and True Negatives (TN).

Figure S2 Relationship between total token consumption and extraction performance.

Figure S3. Impact of visual features on extraction performance.

Figure S4. F1-score and failure examples using automate figure extraction.

Figure S5. Relationship between the number of pages/images per paper and extraction performance across different extraction processes.

Figure S6. Impact of document structural features on extraction performance for PDF inputs.

Figure S7. Extraction performance of the best-performing method across varying matching thresholds.

Figure S8. Distribution of visual features in scatter plots.

Figure S9. Distribution of microscopic image characteristics.

Figure S10. The Distribution of PDF structural features.

Figure S11. Distribution of Publishers and Target Subfigures in the Evaluation Data.

**Table S1. Precision scores of all proposed methods.** The precision scores were calculated from the results of the single-shot extraction and figure-by-figure extraction using Gemini 2.5 Pro with the temperature parameter set to 0.0, as shown in Table 1. Each column, except for “Average,” shows the mean precision across all figures (for figure-by-figure extraction) or all papers (for single-shot extraction). The “Average” column shows the overall mean F1-score calculated from the average F1-scores for each field across all figures and papers. The STD columns represented the standard deviation (STD) across figures or papers. In the performance metric columns, bold text marks the highest value among all methods. The amount of extracted data is provided as a supplementary information.

| Input | Prompt | Protein name | STD of Protein name | Protein conc. | STD of Protein conc. | RNA conc. | STD of RNA conc. | pH | STD of pH | Temperature | STD of temperature | Phase status | STD of phase status | Average | STD of average | Extracted data counts |
| --- | --- | --- | --- | --- | --- | --- | --- | --- | --- | --- | --- | --- | --- | --- | --- | --- |
| Single-shot Extraction | | | | | | | | | | | | | | | | |
| PDF | Minimal + DK + Guide | 0.628 | 0.340 | 0.492 | 0.318 | 0.516 | 0.367 | 0.595 | 0.381 | 0.595 | 0.380 | 0.606 | 0.339 | 0.572 | 0.307 | 707 |
| Figure-by-figure Extraction | | | | | | | | | | | | | | | | |
| Figure | Minimal | 0.705 | 0.392 | 0.628 | 0.452 | 0.882 | 0.209 | 0.041 | 0.200 | 0.034 | 0.169 | 0.432 | 0.408 | 0.453 | 0.170 | 840 |
|  | Minimal + DK | 0.755 | 0.349 | 0.618 | 0.450 | 0.850 | 0.256 | 0.075 | 0.257 | 0.056 | 0.223 | 0.795 | 0.252 | 0.525 | 0.204 | 887 |
|  | Minimal + Guide | 0.683 | 0.392 | 0.800 | 0.317 | 0.859 | 0.228 | 0.851 | 0.258 | 0.796 | 0.327 | 0.729 | 0.296 | 0.786 | 0.220 | 871 |
|  | Minimal + DK + Guide | 0.758 | 0.337 | 0.791 | 0.319 | 0.827 | 0.285 | 0.838 | 0.262 | 0.781 | 0.327 | 0.802 | 0.227 | 0.799 | 0.229 | 888 |
| Figure + Text (C&M) | Minimal | 0.796 | 0.300 | 0.823 | 0.286 | 0.857 | 0.251 | 0.816 | 0.302 | 0.354 | 0.453 | 0.457 | 0.406 | 0.684 | 0.214 | 872 |
|  | Minimal + DK | 0.793 | 0.296 | 0.824 | 0.292 | 0.846 | 0.268 | 0.826 | 0.292 | 0.219 | 0.380 | 0.810 | 0.231 | 0.720 | 0.220 | 893 |
|  | Minimal + Guide | 0.750 | 0.326 | 0.799 | 0.309 | 0.832 | 0.266 | 0.841 | 0.262 | 0.807 | 0.310 | 0.546 | 0.356 | 0.763 | 0.239 | 857 |
|  | Minimal + DK + Guide | **0.832** | **0.289** | **0.835** | **0.289** | 0.871 | 0.233 | **0.876** | **0.233** | **0.842** | **0.289** | **0.819** | **0.226** | **0.846** | **0.232** | 848 |
| Figure + Full Text | Minimal | 0.777 | 0.319 | 0.798 | 0.306 | 0.779 | 0.342 | 0.790 | 0.324 | 0.427 | 0.449 | 0.382 | 0.402 | 0.659 | 0.276 | 925 |
|  | Minimal + DK | 0.784 | 0.321 | 0.830 | 0.303 | 0.845 | 0.279 | 0.811 | 0.324 | 0.305 | 0.433 | 0.826 | 0.246 | 0.734 | 0.245 | 853 |
|  | Minimal + Guide | 0.765 | 0.353 | 0.807 | 0.325 | 0.814 | 0.326 | 0.827 | 0.304 | 0.734 | 0.388 | 0.538 | 0.380 | 0.748 | 0.286 | 824 |
|  | Minimal + DK + Guide | 0.731 | 0.369 | 0.787 | 0.338 | 0.795 | 0.335 | 0.808 | 0.319 | 0.684 | 0.404 | 0.755 | 0.314 | 0.760 | 0.308 | 830 |
| PDF | Minimal | 0.828 | 0.296 | 0.787 | 0.345 | **0.911** | **0.194** | 0.817 | 0.337 | 0.449 | 0.479 | 0.470 | 0.409 | 0.710 | 0.228 | 712 |
|  | Minimal + DK | 0.816 | 0.322 | 0.820 | 0.321 | 0.882 | 0.243 | 0.847 | 0.299 | 0.386 | 0.459 | 0.807 | 0.244 | 0.760 | 0.229 | 771 |
|  | Minimal + Guide | 0.811 | 0.313 | 0.833 | 0.283 | 0.854 | 0.244 | 0.870 | 0.239 | 0.705 | 0.393 | 0.437 | 0.396 | 0.752 | 0.226 | 897 |
|  | Minimal + DK + Guide | 0.788 | 0.321 | 0.828 | 0.279 | 0.859 | 0.211 | 0.859 | 0.240 | 0.814 | 0.292 | 0.769 | 0.243 | 0.820 | 0.225 | 888 |

**Table S2. Recall scores of all proposed methods.** The recall scores were calculated from the results of the single-shot extraction and figure-by-figure extraction using Gemini 2.5 Pro with the temperature parameter set to 0.0, as shown in Table 1. Each column, except for “Average,” shows the mean recall across all figures (for figure-by-figure extraction) or all papers (for single-shot extraction). The “Average” column shows the overall mean recall calculated from the average recall values for each field across all figures and papers. The values following “±” indicate the STD across figures and papers. In the performance metric columns, bold text marks the highest value among all methods. The amount of extracted data is provided as supplemental information. Abbreviations: conc., concentration.

| Input | Prompt | Protein name | STD of Protein name | Protein conc. | STD of Protein conc. | RNA conc. | STD of RNA conc. | pH | STD of pH | Temperature | STD of temperature | Phase status | STD of phase status | Average | STD of average | Extracted data counts |
| --- | --- | --- | --- | --- | --- | --- | --- | --- | --- | --- | --- | --- | --- | --- | --- | --- |
| Single-shot Extraction | | | | | | | | | | | | | | | | |
| PDF | Minimal + DK + Guide | 0.714 | 0.369 | 0.627 | 0.377 | 0.610 | 0.333 | 0.733 | 0.381 | 0.732 | 0.381 | 0.685 | 0.350 | 0.684 | 0.339 | 707 |
| Figure-by-figure Extraction | | | | | | | | | | | | | | | | |
| Figure | Minimal | 0.725 | 0.375 | 0.639 | 0.448 | 0.927 | 0.148 | 0.041 | 0.200 | 0.029 | 0.154 | 0.449 | 0.401 | 0.468 | 0.133 | 840 |
|  | Minimal + DK | 0.821 | 0.329 | 0.652 | 0.455 | **0.945** | **0.138** | 0.080 | 0.271 | 0.049 | 0.207 | **0.889** | **0.157** | 0.573 | 0.154 | 887 |
|  | Minimal + Guide | 0.729 | 0.389 | 0.870 | 0.292 | 0.937 | 0.142 | 0.924 | 0.190 | 0.870 | 0.291 | 0.813 | 0.265 | 0.857 | 0.157 | 871 |
|  | Minimal + DK + Guide | 0.818 | 0.329 | 0.865 | 0.291 | 0.892 | 0.236 | **0.933** | **0.180** | 0.869 | 0.291 | 0.887 | 0.152 | 0.877 | 0.156 | 888 |
| Figure + Text (C&M) | Minimal | 0.845 | 0.262 | 0.880 | 0.245 | 0.913 | 0.192 | 0.878 | 0.263 | 0.367 | 0.461 | 0.495 | 0.415 | 0.730 | 0.185 | 872 |
|  | Minimal + DK | 0.852 | 0.271 | 0.901 | 0.239 | 0.900 | 0.214 | 0.901 | 0.238 | 0.269 | 0.435 | 0.884 | 0.162 | 0.784 | 0.170 | 893 |
|  | Minimal + Guide | 0.821 | 0.292 | 0.874 | 0.261 | 0.906 | 0.194 | 0.916 | 0.187 | 0.875 | 0.260 | 0.636 | 0.370 | 0.838 | 0.193 | 857 |
|  | Minimal + DK + Guide | **0.868** | **0.257** | **0.889** | **0.243** | 0.925 | 0.163 | 0.929 | 0.157 | **0.890** | **0.243** | 0.871 | 0.165 | **0.895** | **0.161** | 848 |
| Figure + Full Text | Minimal | 0.865 | 0.283 | 0.894 | 0.263 | 0.839 | 0.314 | 0.882 | 0.283 | 0.478 | 0.485 | 0.412 | 0.416 | 0.728 | 0.241 | 925 |
|  | Minimal + DK | 0.831 | 0.309 | 0.884 | 0.262 | 0.906 | 0.228 | 0.866 | 0.290 | 0.329 | 0.453 | 0.886 | 0.188 | 0.784 | 0.202 | 853 |
|  | Minimal + Guide | 0.796 | 0.335 | 0.844 | 0.303 | 0.828 | 0.304 | 0.865 | 0.278 | 0.763 | 0.378 | 0.596 | 0.400 | 0.782 | 0.266 | 824 |
|  | Minimal + DK + Guide | 0.780 | 0.355 | 0.853 | 0.299 | 0.846 | 0.300 | 0.874 | 0.272 | 0.753 | 0.394 | 0.813 | 0.270 | 0.820 | 0.264 | 830 |
| PDF | Minimal | 0.746 | 0.329 | 0.752 | 0.364 | 0.815 | 0.269 | 0.747 | 0.351 | 0.403 | 0.455 | 0.434 | 0.382 | 0.650 | 0.273 | 712 |
|  | Minimal + DK | 0.758 | 0.333 | 0.813 | 0.321 | 0.853 | 0.254 | 0.816 | 0.302 | 0.364 | 0.438 | 0.778 | 0.245 | 0.730 | 0.233 | 771 |
|  | Minimal + Guide | 0.796 | 0.309 | 0.848 | 0.265 | 0.872 | 0.219 | 0.887 | 0.213 | 0.725 | 0.385 | 0.465 | 0.398 | 0.766 | 0.205 | 897 |
|  | Minimal + DK + Guide | 0.781 | 0.328 | 0.853 | 0.274 | 0.881 | 0.218 | 0.886 | 0.235 | 0.844 | 0.296 | 0.793 | 0.238 | 0.840 | 0.218 | 888 |

**Table S3. Results of paired Wilcoxon signed-rank tests comparing the best-performing method (Fig. + Text (C&M) with Minimal + DK + Guide) against other extraction methods of the figure-by-figure extraction.** The tests were performed based on the average F1-score across six experimental conditions for each target subfigure. "Diff" represents the difference in average F1-scores. The p-values were adjusted for multiple comparisons using the Holm method. Statistical significance was defined as p < 0.05 (Significant: TRUE).

| Comparison to the best method  [Fig. + Text (C&M), Minimal + DK + Guide] | | Diff | p-value | Significant |
| --- | --- | --- | --- | --- |
| Figure | Minimal | 0.196 | 4.65 × 10^-15^ | TRUE |
|  | Minimal + DK | 0.127 | 9.31 × 10^-09^ | TRUE |
|  | Minimal + Guide | 0.122 | 6.13 × 10^-04^ | TRUE |
|  | Minimal + DK + Guide | 0.027 | 3.09 × 10^-02^ | TRUE |
| Fig. + Text (C&M) | Minimal | 0.390 | 5.33 × 10^-07^ | TRUE |
|  | Minimal + DK | 0.309 | 4.89 × 10^-08^ | TRUE |
|  | Minimal + Guide | 0.043 | 4.15 × 10^-03^ | TRUE |
| Fig. + Full Text | Minimal | 0.163 | 5.85 × 10^-07^ | TRUE |
|  | Minimal + DK | 0.102 | 7.71 × 10^-04^ | TRUE |
|  | Minimal + Guide | 0.102 | 3.56 × 10^-04^ | TRUE |
|  | Minimal + DK + Guide | 0.082 | 1.51 × 10^-03^ | TRUE |
| PDF | Minimal | 0.152 | 2.89 × 10^-07^ | TRUE |
|  | Minimal + DK | 0.115 | 4.56 × 10^-05^ | TRUE |
|  | Minimal + Guide | 0.068 | 4.58 × 10^-05^ | TRUE |
|  | Minimal + DK + Guide | 0.041 | 2.06 × 10^-01^ | FALSE |

**Table S4. Average F1-scores from temperature parameter optimization for Gemini 2.5 Pro**. Comparison of average F1-scores evaluated for Gemini 2.5 Pro across different temperature settings (0.0–0.5) using figure-by-figure extraction, Figure + Text (C&M) input, and Minimal + DK + Guide prompt. Each extraction experiment was repeated three times (N = 3), and the average F1-scores from these repetitions are reported in the “AVG.” column. The STD values across the figures are reported in the “STD” column.

|  | AVG. | STD |
| --- | --- | --- |
| Temp. = 0.0 | 0.831 | 0.0083 |
| Temp. = 0.1 | **0.836** | 0.0125 |
| Temp. = 0.2 | 0.826 | 0.0107 |
| Temp. = 0.5 | 0.829 | 0.0043 |

**Table** S5**. Computational cost analysis for each extraction configuration.** All data were calculated based on the results obtained using Gemini 2.5 Pro across the entire evaluation dataset (n = 49 images for figure-by-figure extraction; n = 20 papers for single-shot extraction). The "Total tokens" column represents the sum of "Input tokens," "Output tokens," and "Figure or PDF tokens," providing an estimate of the overall computational resources required for each processing method.

|  | Input Format | Prompt | Total tokens | Input tokens | Output tokens | Figure or PDF tokens |
| --- | --- | --- | --- | --- | --- | --- |
| Figure-by-figure Extraction | Figure | Minimal | 108,108 | 15363 | 79329 | 13416 |
|  |  | Minimal + DK | 146,523 | 42611 | 90496 | 13416 |
|  |  | Minimal + Guide | 149,224 | 53905 | 81903 | 13416 |
|  |  | Minimal + Guide + DK | 177,231 | 77981 | 85834 | 13416 |
|  | Fig. + Text (C&M) | Minimal | 373,584 | 245454 | 114714 | 13416 |
|  |  | Minimal + DK | 392,429 | 272702 | 106311 | 13416 |
|  |  | Minimal + Guide | 389,450 | 283997 | 92037 | 13416 |
|  |  | Minimal + Guide + DK | 411,878 | 308073 | 90389 | 13416 |
|  | Fig. + Text (Total) | Minimal | 1,350,979 | 1221280 | 116283 | 13416 |
|  |  | Minimal + DK | 1,364,083 | 1248528 | 102139 | 13416 |
|  |  | Minimal + Guide | 1,361,283 | 1259667 | 88200 | 13416 |
|  |  | Minimal + Guide + DK | 1,389,369 | 1283899 | 92054 | 13416 |
|  | PDF | Minimal | 540,072 | 15363 | 91269 | 433,440 |
|  |  | Minimal + DK | 570,526 | 42611 | 94475 | 433,440 |
|  |  | Minimal + Guide | 578,116 | 53905 | 90771 | 433,440 |
|  |  | Minimal + Guide + DK | 540,375 | 40506 | 66429 | 433,440 |
| Single-shot Extraction | PDF | Minimal + Guide + DK | 540,072 | 15363 | 91269 | 433,440 |

**Table S6. Average and total extraction costs per figure for each MLLMs.** The computational costs were calculated for the figure-by-figure extraction experiments using Figure + Text (C&M) as the input format and Minimal + Guide + DK as the input prompt. The average token consumption per processing unit was 5,388 tokens. Based on this value, the cost in USD for each model was estimated and summarized in the table. The Average cost represents the mean cost per processing unit, whereas the total cost indicates the cumulative cost of processing 20 papers. The Claude Sonnet models share an identical pricing structure and are therefore presented in a single column.

|  | Gemini 2.5 Pro | Gemini 2.0 Flash | Gemini 1.5 Flash | GPT-5 | GPT-4.1 | GPT-4o | Claude Sonnet series |
| --- | --- | --- | --- | --- | --- | --- | --- |
| Average cost (USD) | 0.00724 | 0.00362 | 0.00087 | 0.08387 | 0.01158 | 0.05493 | 0.01737 |
| Total cost (USD) | 0.40522 | 0.20261 | 0.04863 | 4.69675 | 0.64835 | 3.07588 | 0.97253 |

**Table S7. Comparison of extraction performance between scatter plots and microscopic images.** The average F1-scores under each prompt condition: Minimal, Minimal + DK, Minimal + Guide, and Minimal + DK + Guide when using Gemini 2.5 Pro as the base MLLM and Figure + Text (C&M) as the input format. Abbreviations: temp., temperature.

| Input | MLLM | Prompt | Scatter plots | Microscopic images |
| --- | --- | --- | --- | --- |
| Figure + Text (C&M) | Gemini 2.5 Pro  (Temp. = 0.0) | Minimal | 0.803 | 0.660 |
|  |  | Minimal + DK | 0.791 | 0.707 |
|  |  | Minimal + Guide | 0.838 | 0.776 |
|  |  | Minimal + DK + Guide | 0.848 | 0.849 |
|  | Gemini 2.5 Pro  (Temp. = 0.1) | Minimal + DK + Guide | 0.913 | 0.829 |

**Table S8. Correspondence between papers and reference numbers in the evaluation dataset.** The table lists the papers used in the evaluation and their corresponding reference numbers in the main text.

| Reference number | Reference |
| --- | --- |
| 36 | Molliex, A. et al. Phase separation by low complexity domains promotes stress granule assembly and drives pathological fibrillization. *Cell* **163**, 123–133 (2015). 10.1016/j.cell.2015.09.015, PubMed: 26406374. |
| 37 | Lin, Y., Protter, D. S. W., Rosen, M. K. & Parker, R. Formation and maturation of phase-separated liquid droplets by RNA-binding proteins*. Mol. Cell* **60**, 208–219 (2015). 10.1016/j.molcel.2015.08.018, PubMed: 26412307. |
| 38 | Zhang, H. et al*.* RNA controls PolyQ protein phase transitions. *Mol. Cell* **60**, 220–230 (2015). 10.1016/j.molcel.2015.09.017, PubMed: 26474065. |
| 39 | Smith, J. et al. Spatial patterning of P granules by RNA-induced phase separation of the intrinsically disordered protein MEG-3 . *eLife* **5**, e21337 (2016). 10.7554/eLife.21337, PubMed: 27914198. |
| 40 | Lin, Y., Currie, S. L. & Rosen, M. K. Intrinsically disordered sequences enable modulation of protein phase separation through distributed tyrosine motifs. *J. Biol. Chem.* **292**, 19110–19120 (2017). 10.1074/jbc.M117.800466, PubMed: 28924037. |
| 41 | Wei, M. T. et al. Phase behaviour of disordered proteins underlying low density and high permeability of liquid organelles. *Nat. Chem.* **9**, 1118–1125 (2017). 10.1038/nchem.2803, PubMed: 29064502. |
| 42 | Protter, D. S. W. et al. Intrinsically disordered regions can contribute promiscuous interactions to RNP granule assembly. *Cell Rep.* **22**, 1401–1412 (2018). 10.1016/j.celrep.2018.01.036, PubMed: 29425497. |
| 43 | Maharana, S. et al. RNA buffers the phase separation behavior of prion-like RNA-binding proteins . *Science* **360**, 918–921 (2018). 10.1126/science.aar7366, PubMed: 29650702. |
| 44 | Langdon, E. M. et al. mRNA structure determines specificity of a polyQ-driven phase separation. *Science* **360**, 922–927 (2018). 10.1126/science.aar7432, PubMed: 29650703. |
| 45 | Hofweber, M. et al. Phase separation of FUS is suppressed by its nuclear import receptor and arginine methylation. *Cell* **173**, 706–719.e13 (2018). 10.1016/j.cell.2018.03.004, PubMed: 29677514. |
| 46 | Kroschwald, S. et al. Different material states of Pub1 condensates define distinct modes of stress adaptation and recovery. *Cell Rep.* **23**, 3327–3339 (2018). 10.1016/j.celrep.2018.05.041, PubMed: 29898402. |
| 47 | Wang, M. et al*.* Stress-induced low complexity RNA activates physiological amyloidogenesis. *Cell Rep.* **24**, 1713–1721.e4 (2018). 10.1016/j.celrep.2018.07.040, PubMed: 30110628. |
| 48 | Tsang, B. et al. Phosphoregulated FMRP phase separation models activity-dependent translation through bidirectional control of mRNA granule formation. *Proc. Natl Acad. Sci. U. S. A.* **116**, 4218–4227 (2019). 10.1073/pnas.1814385116, PubMed: 30765518. |
| 49 | Kang, J., Lim, L., Lu, Y. & Song, J. A unified mechanism for LLPS of ALS/FTLD-causing FUS as well as its modulation by ATP and oligonucleic acids. *PLOS Biol.* **17**, e3000327 (2019). 10.1371/journal.pbio.3000327, PubMed: 31188823. |
| 50 | Tari, M. et al. U2AF^65^ assemblies drive sequence-specific splice site recognition. *EMBO Rep.* **20**, e47604 (2019). 10.15252/embr.201847604, PubMed: 31271494. |
| 51 | Ries, R. J. et al. m6A enhances the phase separation potential of mRNA. *Nature* **571**, 424–428 (2019). 10.1038/s41586-019-1374-1, PubMed: 31292544. |
| 52 | Niaki, A. G. et al*.* Loss of dynamic RNA interaction and aberrant phase separation induced by two distinct types of ALS/FTD-linked FUS mutations. *Mol. Cell* **77**, 82–94.e4 (2020). 10.1016/j.molcel.2019.09.022, PubMed: 31630970. |
| 53 | Huo, X. et al*.* The nuclear matrix protein SAFB cooperates with major satellite RNAs to stabilize heterochromatin architecture partially through phase separation. *Mol. Cell* **77**, 368–383.e7 (2020). 10.1016/j.molcel.2019.10.001, PubMed: 31677973. |
| 54 | Chen, H. et al*.* Liquid–liquid phase separation by SARS-CoV-2 nucleocapsid protein and RNA. *Cell Res.* **30**, 1143–1145 (2020). 10.1038/s41422-020-00408-2, PubMed: 32901111. |
| 55 | Jack, A. et al*.* SARS-CoV-2 nucleocapsid protein forms condensates with viral genomic RNA. *PLOS Biol.* **19**, e3001425 (2021). 10.1371/journal.pbio.3001425, PubMed: 34634033. |


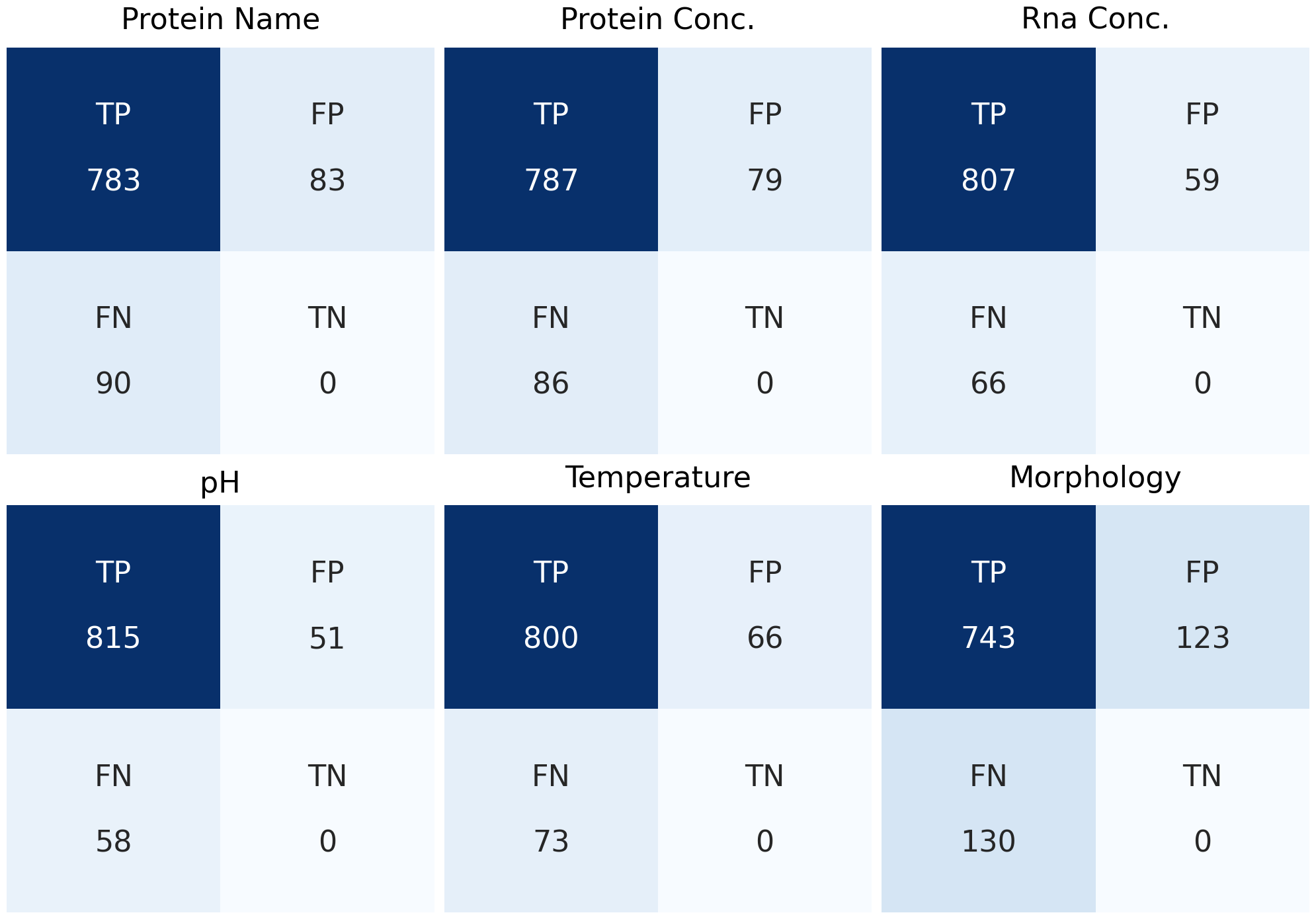


**Figure S1. Confusion matrices showing the counts of True Positives (TP), False Positives (FP), False Negatives (FN), and True Negatives (TN).** The matrices were generated based on the matching results of the best-performing combination (Gemini 2.5 Pro, Figure-by-figure Extraction, Fig. + Text (C&M), Minimal + DK + Guide) to show the number of data points for each class.


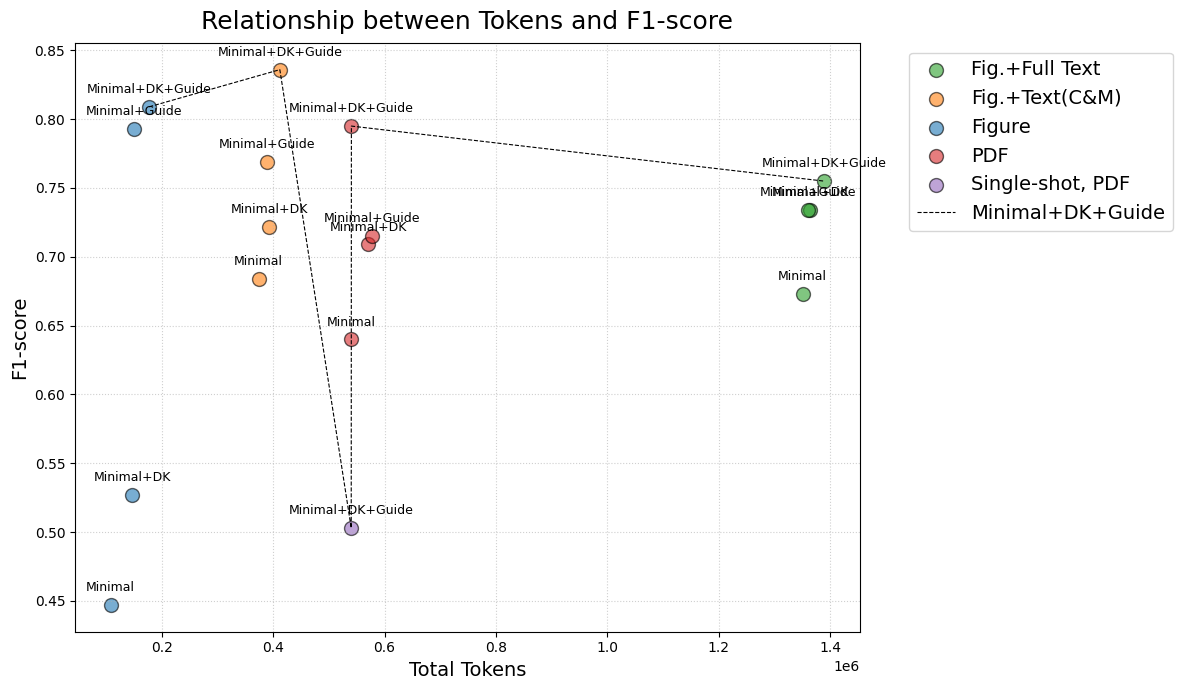


**Figure S2 Relationship between total token consumption and extraction performance.** The figure shows the relationship between operational costs and the F1-score for various input formats and prompt configurations. The analysis was conducted using 20 papers (including 49 target figures) processed with Gemini 2.5 Pro. Colors represent different input and processing method groups, including Figure only (blue), Figure + Text (C&M) (orange), PDF (red), Figure + Full Text (green) for figure-by-figure extraction, and PDF for single-shot extraction (purple). Labels indicate prompt types, and the dashed line tracks the performance of the "Minimal + DK + Guide" prompt across the different inputs and processing methods.


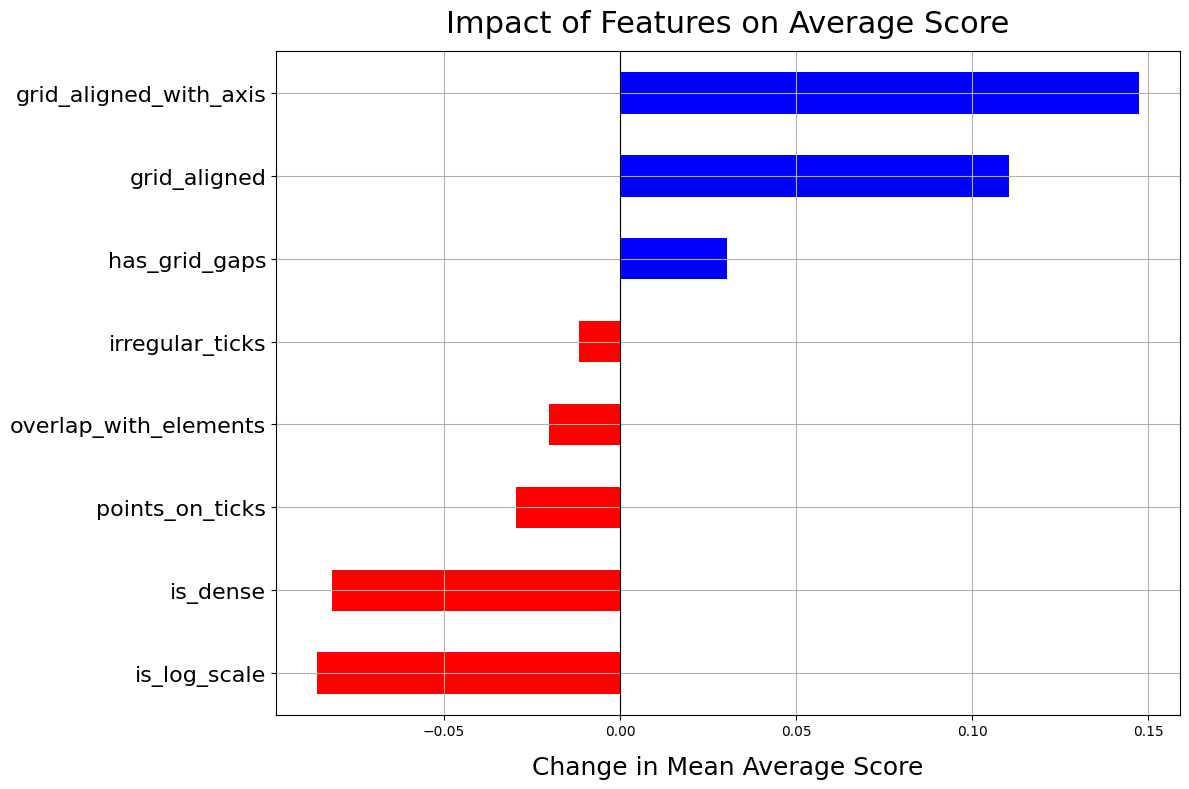


**Figure S3. Impact of visual features on extraction performance.** The extraction performance of each scatter plot, based on the best-performing method, is categorized according to the presence or absence of features within the graph. Each bar represents the change in the mean F1-score associated with specific visual characteristics of the figures. "grid_aligned_with_axis" refers to a grid arrangement aligned with the intersections of x- and y-axis ticks, while "grid_aligned" refers to a general grid-like layout. "has_grid_gaps" denotes missing segments within the grid arrangement, and "irregular_ticks" refers to non-uniform tick intervals. "overlap_with_elements" indicates cases where data points overlap with other chart elements such as legends, and "points_on_ticks" indicates whether data points are placed directly on the axes. "is_dense" describes instances where data points overlap or are in contact with each other, and "is_log_scale" refers to axes using a logarithmic scale. Blue bars indicate features that contribute positively to extraction performance, whereas red bars indicate features that negatively impact performance.

**Figure S4. F1-score and failure examples using automate figure extraction.** To verify whether figure extraction can be automated, we conducted an experiment to extract figure numbers from the PDFs of the 20 evaluation papers included in the evaluation dataset. Similar to single-shot extraction, we input PDFs with added figure number extraction prompts into the MLLM and extracted all relevant figure numbers in bulk. The prompt consisted of two elements: first, requesting an enumeration of all figure numbers within the paper; second, requesting descriptions for each figure, verifying whether they met the extraction criteria, and then compiling a list of all figure numbers satisfying all criteria. Prompt details are documented in the sixth section of the “additional_file2.docx” file. The experiment was conducted using Gemini 2.5 Pro with a temperature parameter set to 0. All the other parameters were maintained at their default values. The extracted results were compared with the target figure numbers in the evaluation dataset. Matches were counted as true positives and the F1 score was calculated. The average F1 score across all the papers served as the final evaluation metric. (A) Average F1-scores for extraction of relevant figure numbers from each paper. The rightmost bar indicates the average across all papers, and the error bars indicate the STD of the average F1-score between each paper. (B) Examples of false positives. Specifically, Fig. S4A^36^ and Fig. 2B^46^ does not contain RNA, Fig. S3D^47^ does not show LLPS, and Fig. S1C^48^ varied the time parameter, which was outside the scope of the extraction. The reference numbers correspond to citations in the main text and Table S1. Therefore, these figures were not included as targets for extraction in this study. Image in Fig. S4A^36^ were reproduced with permission from the paper^36^. Image in Fig. 2B^46^ was reproduced with permission from the paper^46^. Image in Fig. S3D^47^ was reproduced with permission from the paper^47^. Image in Fig. S1C^48^ were reproduced with permission from the paper^48^.

**Figure S5. Relationship between the number of pages/images per paper and extraction performance across different extraction processes.** All processes are based on experimental results using Gemini 2.5 Pro. The figure for Single-shot Extraction uses extraction performance per paper as data points, while Figure-by-figure uses extraction performance per image as data points. For figure-by-figure extraction, the input is PDF, and the prompt is Minimal + DK + Guide. (A, C). The relationship between the number of pages per paper and the F1-score is shown for each target experimental information. Red lines indicate linear regression with 95% confidence intervals. The value of “r” in the legend denotes the correlation coefficient. (B, D). The relationship between the number of target images in a paper and the F1-score is shown for each target experimental information. Blue lines indicate linear regression with 95% confidence intervals. The value of “r” in the legend denotes the correlation coefficient.


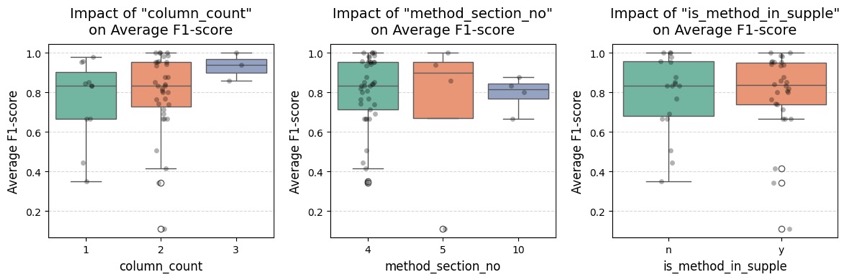


**Figure S6. Impact of document structural features on extraction performance for PDF inputs.** The box plots show the average F1-scores for the PDF input series under the figure-by-figure extraction method using the Minimal + DK + Guide prompt. The analysis examined the influence of three structural characteristics: the number of columns in the document layout (column_count), the specific section where the methods were described (method_section_no), and whether the methodology was in the supplementary information (is_method_in_supple). The number in “method_section_no” represents the appearance number of the methods section; papers included in “10” describe their methods in the supplement.


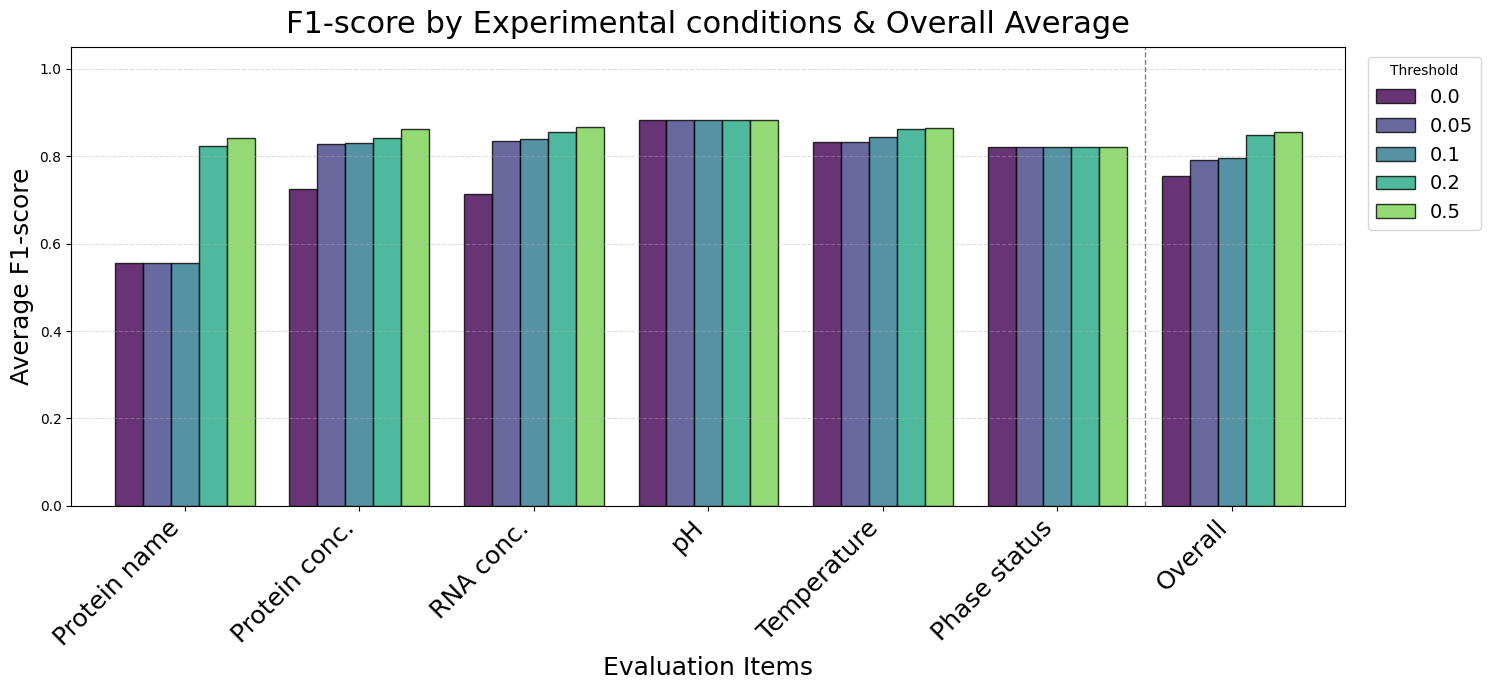


**Figure S7. Extraction performance of the best-performing method across varying matching thresholds.** The Average F1-score across the target figure was calculated by varying the matching threshold between 0.0 and 0.5 for the best-performing method. The best performing method consisted of Gemini 2.5 Pro using figure-by-figure extraction with the Figure + Text (C&M) input pattern and the Minimal + DK + Guide prompt. The results are displayed for each experimental condition, and the overall average is shown in the rightmost group.


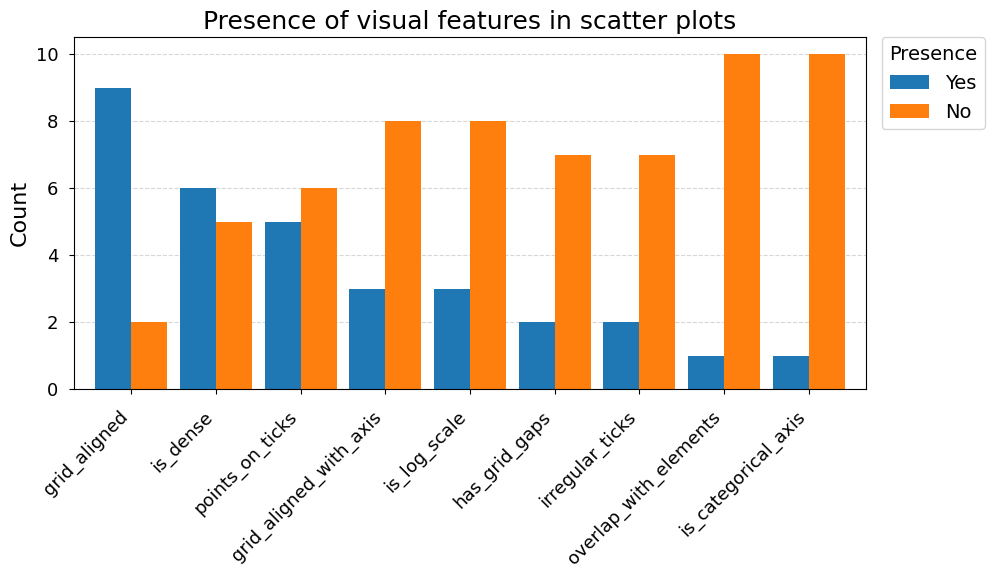


**Figure S8. Distribution of visual features in scatter plots.** The presence or absence of specific visual characteristics in the analyzed scatter plots is summarized. Each feature corresponds to the definitions used in Figure S3. “grid_aligned_with_axis” refers to a grid arrangement aligned with the intersections of x- and y-axis ticks, whereas “grid_aligned” denotes a general grid-like layout. “has_grid_gaps” indicates missing segments within the grid structure, and “irregular_ticks” refers to non-uniform tick intervals. “overlap_with_elements” represents cases in which data points overlap with other graphical elements, such as legends, and “points_on_ticks” indicates whether data points are positioned directly on axis ticks. “is_dense” describes plots with closely spaced or overlapping data points, and “is_log_scale” refers to axes displayed on a logarithmic scale. Bars represent the number of figures in which each feature is present or absent.

**Figure S9. Distribution of microscopic image characteristics.** (A) Distribution of x–y axis elements in microscopic image subfigures. “Other” includes variables such as pH, temperature, and salt concentration. (B) Distribution of microscopic image arrangements within a single subfigure. “Grid” indicates a matrix-like layout, whereas “Single row” indicates a linear arrangement. (C) Distribution of background–object color combinations in microscopic images. In each pair, the color to the left of “|” represents the background, and the color to the right represents the object.


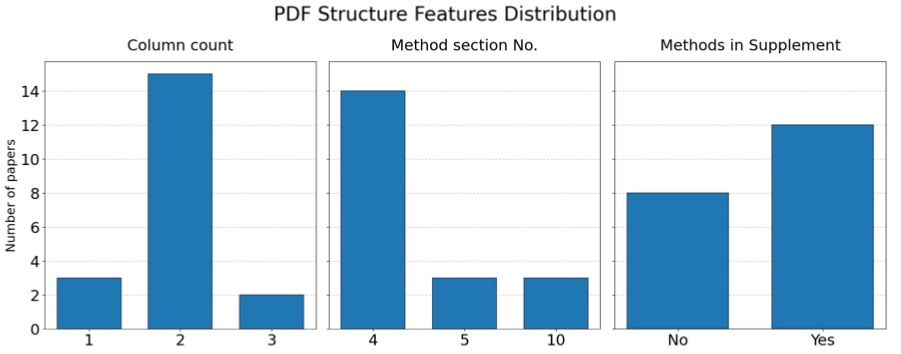


**Figure S10. The Distribution of PDF structural features.** Distribution of PDF structure characteristics across the analyzed papers, including the number of columns per page (left), the section number at which the Methods section appears (center), and the presence or absence of supplementary Methods sections (right). The y-axis indicates the number of papers in each category.

**Figure S11. Distribution of Publishers and Target Subfigures in the Evaluation Data.** (A) Details of the publishers of the papers used in the evaluation data are shown. (B) The distribution of the target subfigure counts included in each paper is shown.
